# Supplementary material for: Pancreatic glycoprotein 2 is a first line of defense for mucosal protection in intestinal inflammation
Source: Nat Commun. 2021 Feb 16;12:1067. doi: 10.1038/s41467-021-21277-2 (PMC7887276; doi:10.1038/s41467-021-21277-2)
Supplement: Supplementary file 1 — Supplementary Information [file 41467_2021_21277_MOESM1_ESM.pdf]

Supplementary Information for

Pancreatic Glycoprotein 2 is a First Line of Defense for Mucosal Protection in Intestinal Inflammation

Kurashima Y et al

Supplementary Figure 1

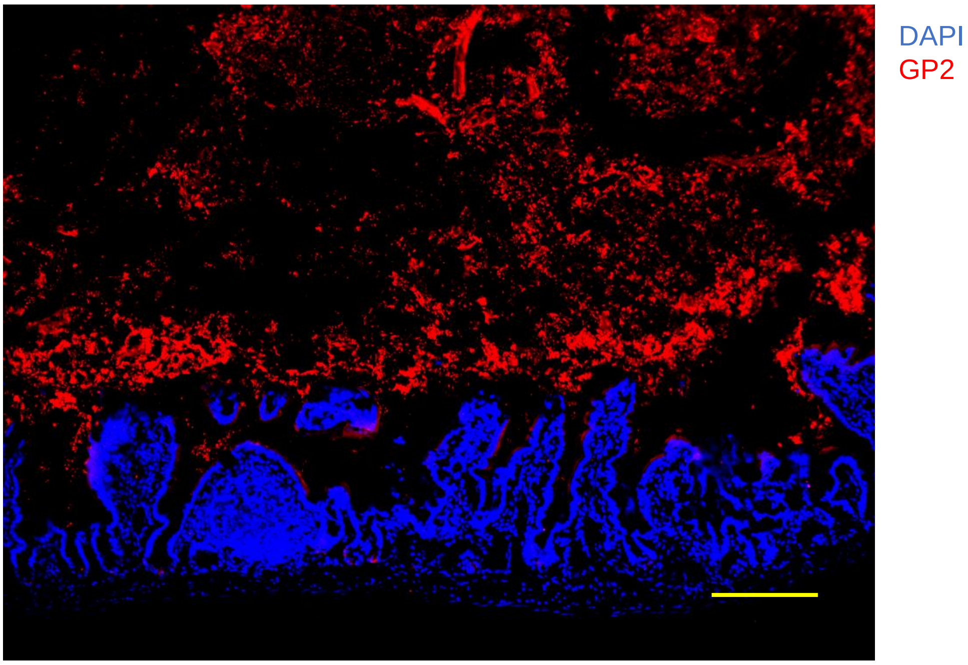

Dissemination of GP2 in the intestinal lumen.

Immunohistochemical analysis of GP2 distribution in the gastrointestinal tract with luminal contents were shown. GP2 (red), DAPI (blue). Scale bars, 100 μm. Data are representative of at least three independent experiments.

Supplementary Figure 2

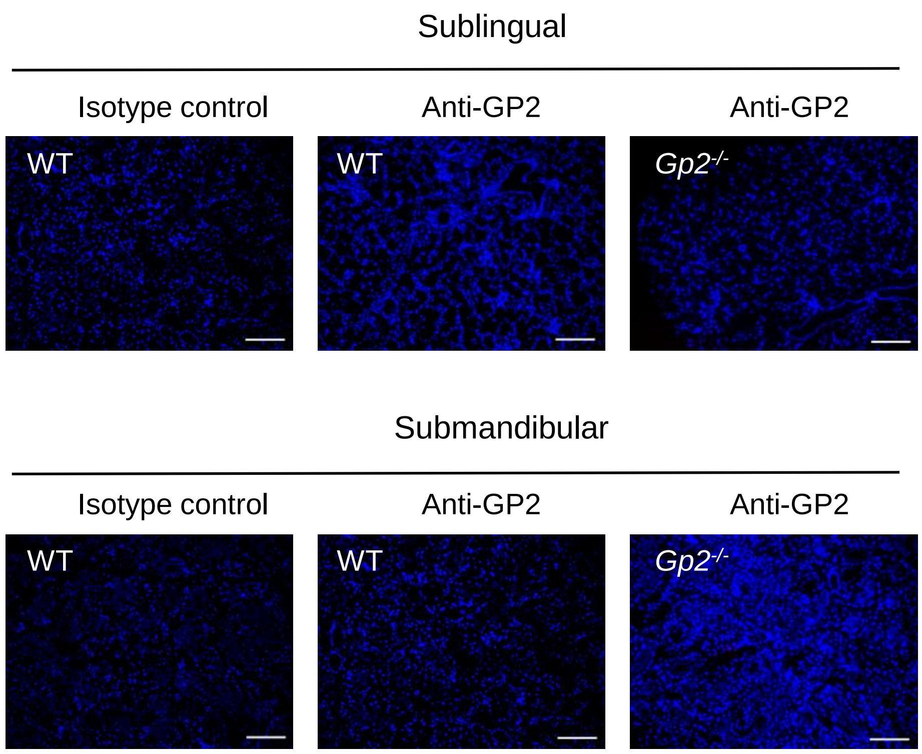

No observation of GP2 in the salivary glands.

Immunohistochemical analysis of GP2 distribution in the sublingual and submandibular areas of an intact mouse. Representative data of three independent experiments were shown. GP2 (red) and DAPI (blue). Scale bars, 100 μm.

Supplementary Figure 3

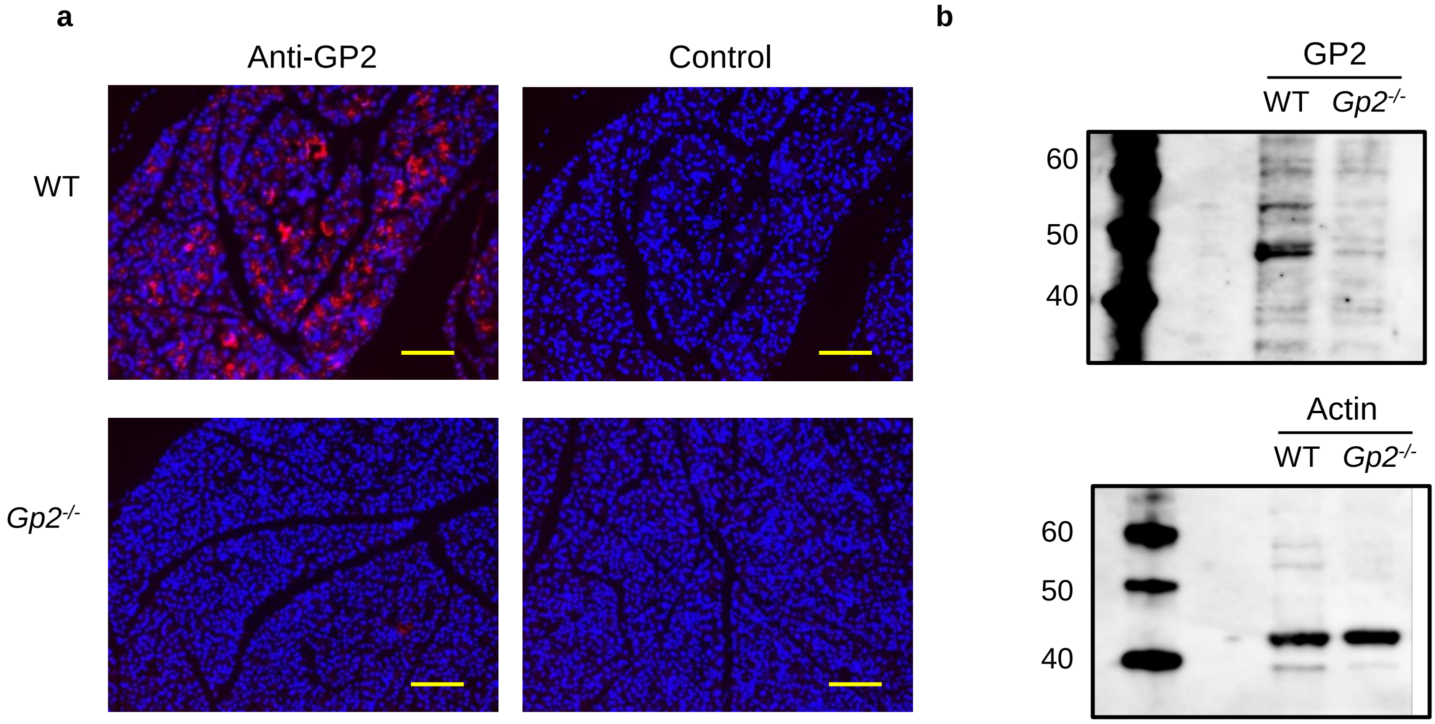

Detection of pancreatic GP2.

a, Pancreas from WT and *GP2*$^{-/-}$ mice were stained with or without anti-GP2 polyclonal antibody. Scale bars: 100 μm. Data are representative of two independent experiments. **b**, Expression of GP2 (Thermo Fisher Scientific, polyclonal, #PA5-88542, 1:250) and actin (Merck Millipore, clone C4, #MAB1501R, 1:3000) in pancreas cells was examined by western blot analysis. Data are representative of two independent experiments.

Supplementary Figure 4

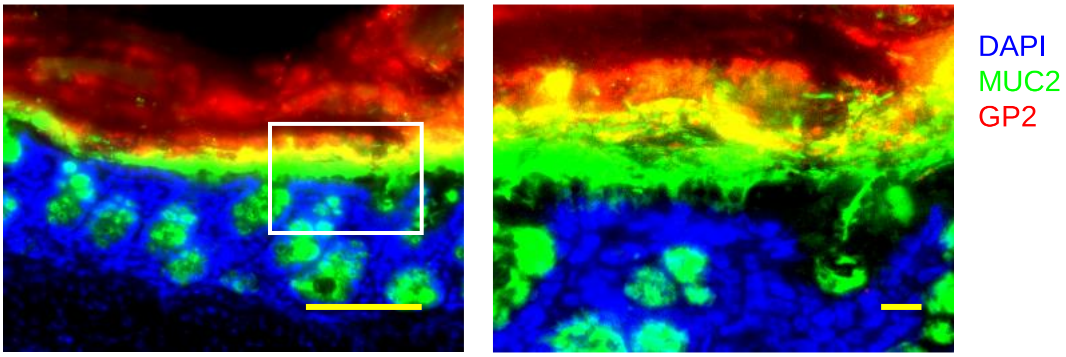

Localization of pancreatic GP2 in the mucus layer.

Immunohistochemical analysis of GP2 distribution in the mucus layer was performed. Representative data of four independent experiments are shown. GP2 (red), MUC2 (green), and DAPI (blue). Scale bars, 100 μm (left panel) and 20 μm (right panel).

Supplementary Figure 5

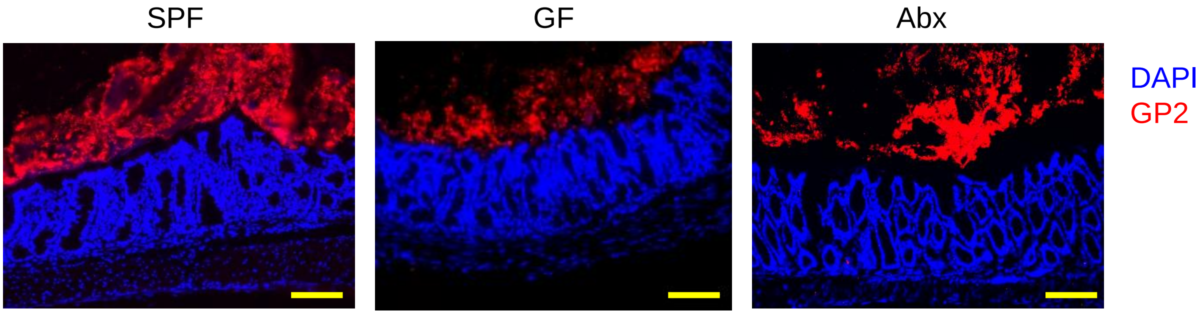

Detection of luminal GP2 in germ free conditions.

GP2 distribution in the colon in SPF, germ-free (GF), antibiotics (Abx) treated mice are shown. Representative data of three independent experiments are shown. GP2 (red), DAPI (blue). Scale bars, 100 μm.

Supplementary Figure 6

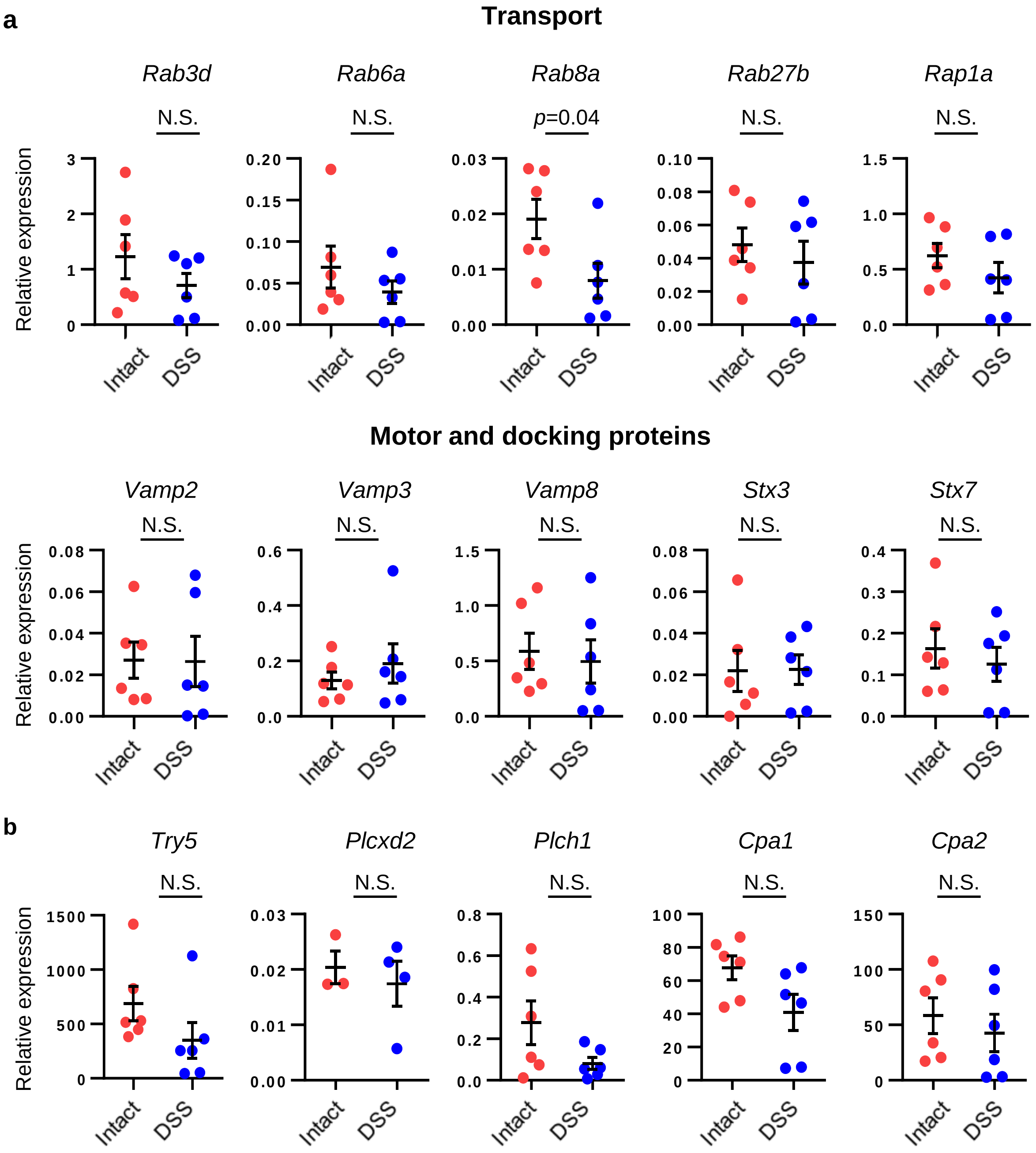

Granular release of pancreatic GP2 in colitis.

a, Expressions of genes encoding granular transport proteins (*Rab3d*, *Rab61*, *Rab8a*, *Rab27b*, *Rap1a*) and motor and docking proteins (*Vamp2*, *Vamp3*, *Vamp8*, *Stx3*, *Stx7*) in the pancreas from intact and DSS treated mice were determined by qPCR (n=6). N.S. indicates not significant (two-tailed unpaired *t*-test). Data are presented as mean values +/- SEM. **b**, Expressions of genes encoding enzymes involved in glycosylphosphatidylinositol anchor cleavage (*Try5*, *Plcxd2*, *Plch1*, *Cpa1*, and *Cpa2*) were determined by qPCR. Relative expression of mRNA was determined by quantitative RT-PCR and normalized to the expression of *Gapdh* (n=6). N.S. indicates not significant (two-tailed unpaired *t*-test). Data are presented as mean values +/- SEM. Source data are provided as a Source Data file.

Supplementary Figure 7

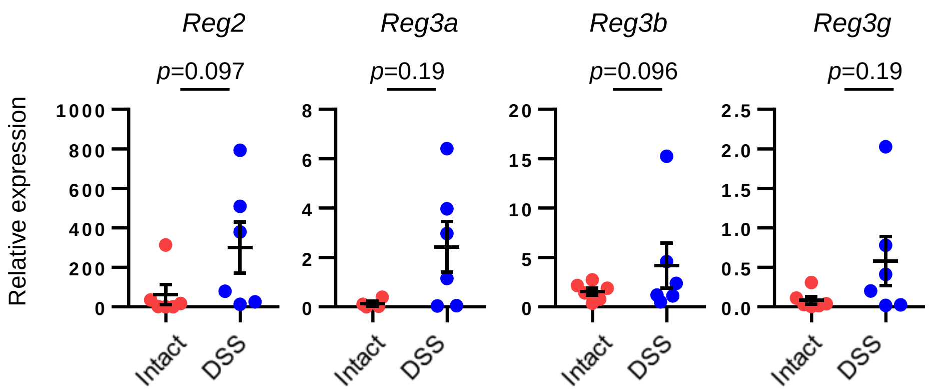

Antibiotic protein expressions in pancreas.

a, Expressions of genes encoding Reg proteins (*Reg2*, *Reg3a*, *Reg3b*, *Reg3g*) in the pancreas from intact and DSS treated mice were determined by qPCR. Relative expression of mRNA was determined by quantitative RT-PCR and normalized to the expression of *Gapdh* (n=6). Data are presented as mean values +/- SEM. Source data are provided as a Source Data file.

Supplementary Figure 8

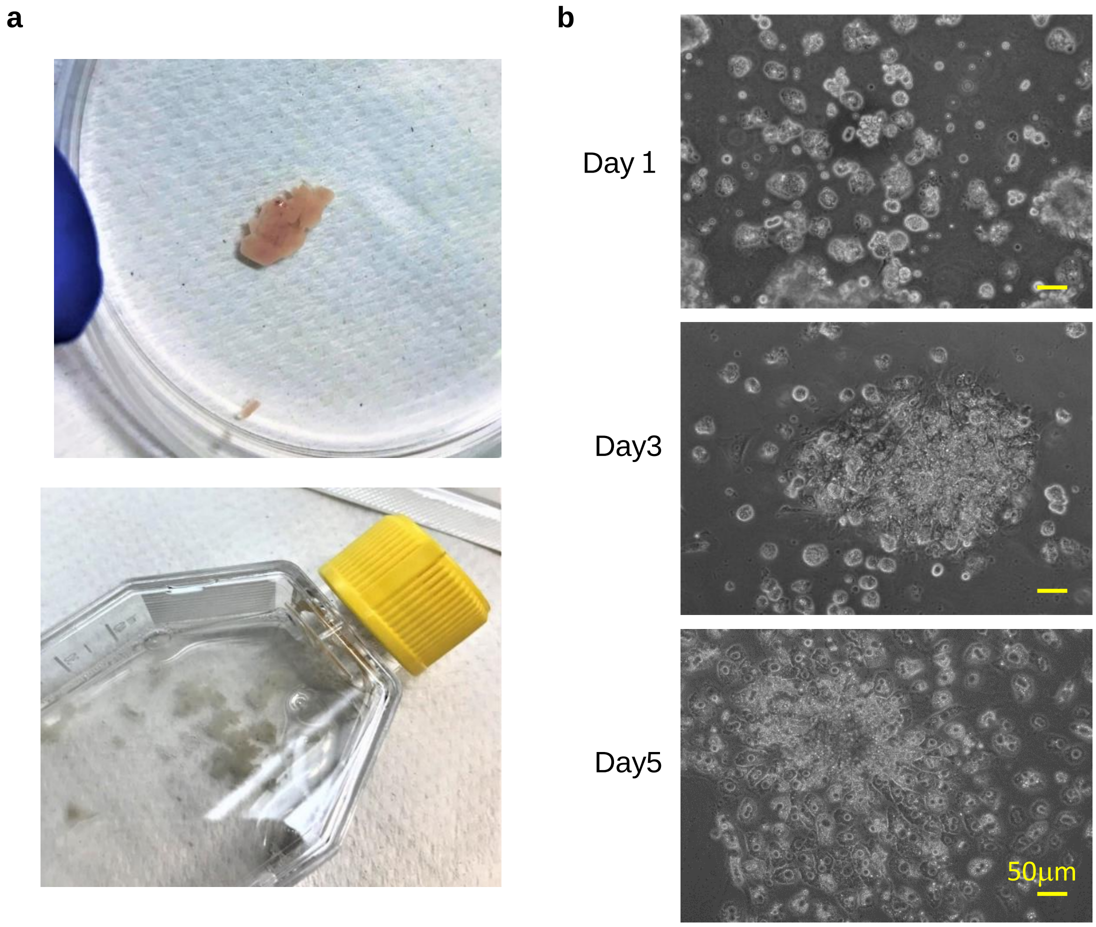

***In vitro* culture of pancreatic acinar cells.**

a, Pancreas was isolated from mice (upper panel) and enzymatically digested as described in lower panel. **b**, Cultured acinar cells in day1, 3, 5 were shown. Data are representative of four independent experiments.

Supplementary Figure 9

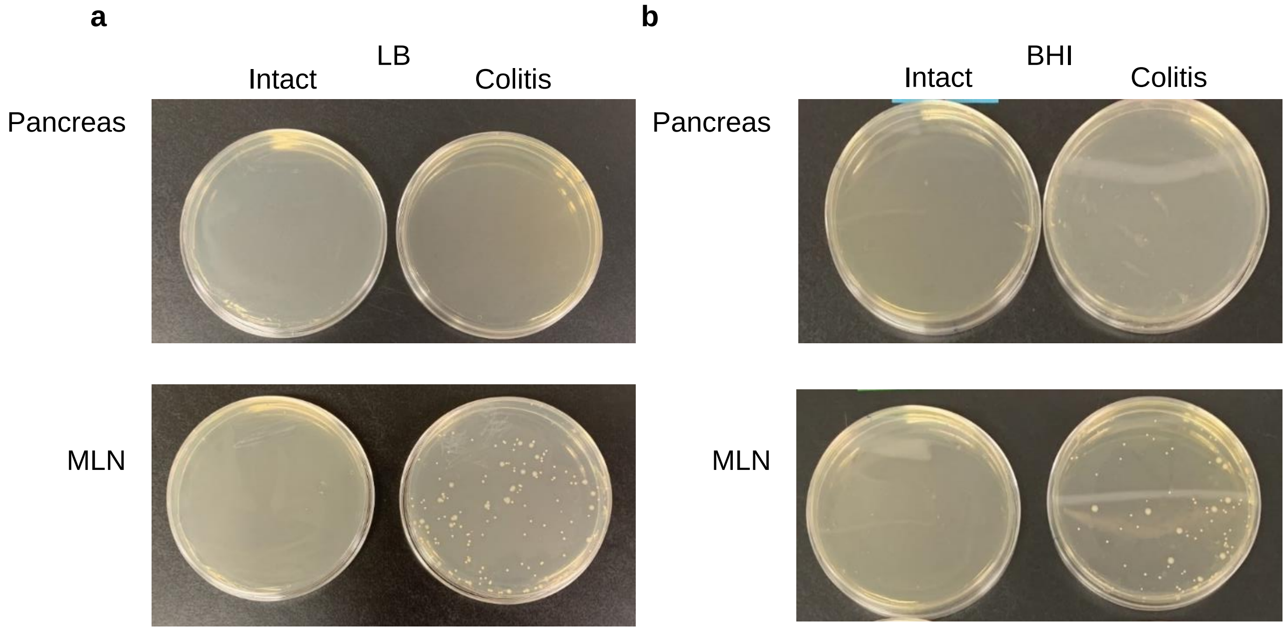

Bacterial translocation in mesenteric lymph nodes and pancreas.

Mesenteric lymph nodes (MLN) and pancreas were isolated from intact and DSS-treated mice, enzymatically digested, and then cultured on LB (**a**) and BHI (**b**) plates. Representative photographs are shown.

Supplementary Figure 10

a

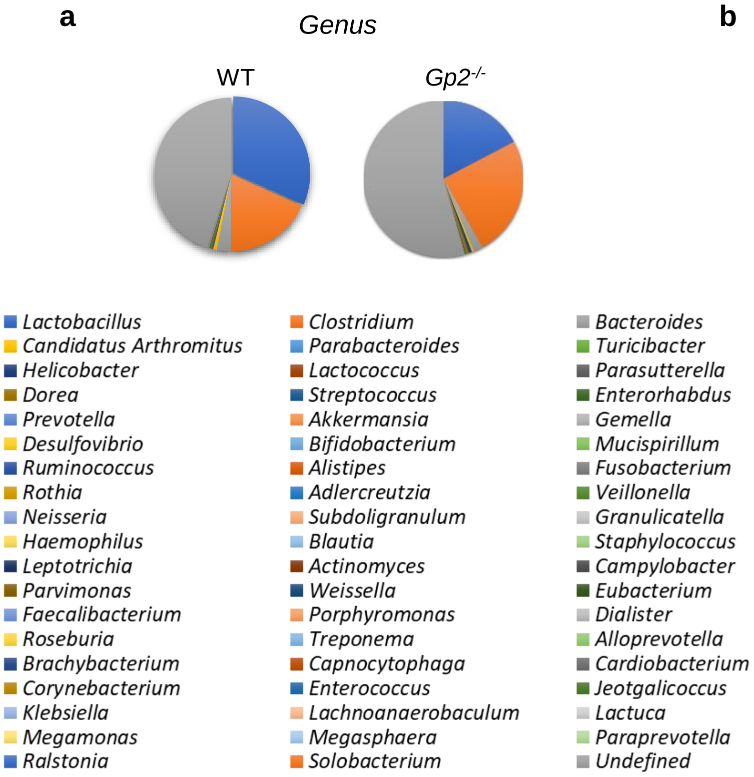

b

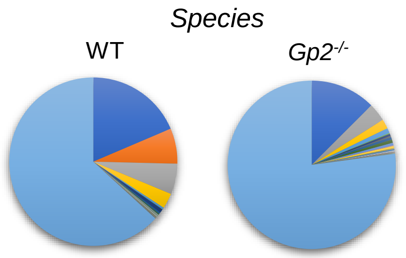

Species	WT	KO	p value (t-test)
Helicobacter sp. MIT 01-6451	3.75	15.8	0.034150233
Clostridium sp. ASF356	0.5	11	0.037486415
Bacteroides acidifaciens	88.75	54.8	0.040344507
Clostridium fusiformis	5.5	19	0.058353989
Clostridium sp. A9	0	0.6	0.070483997
Clostridium sp. Culture-46	0	1.2	0.108700951
Clostridium sp. ASF502	8.75	15.8	0.111258841
Clostridium sp. Culture Jar-8	0.75	6.8	0.145524071
Gemella sanguinis	0	0.4	0.177807808
Parabacteroides sp. D13	0	0.4	0.177807808
Prevotella nanceiensis	0	0.4	0.177807808
Veillonella dispar	0	0.4	0.177807808
Fusobacterium periodonticum	0.5	0	0.181690114
Prevotella DO039	0.5	0	0.181690114
Lactobacillus intestinalis	203.75	0	0.188858302
Streptococcus infantis	0.5	1	0.281240276
Clostridium sp. cTPY-12	0.75	3	0.290993186
Prevotella melaninogenica	1.25	0.4	0.303394369
Clostridium sp. Clone-45	0.25	4	0.320006626
Lactobacillus sp. Is79	1.5	4.8	0.332588568
Undefined	1896.25	2313.6	0.349536266
Prevotella histicola	0.25	0.6	0.351487074
Clostridium sp. Culture-57	2.25	4	0.370483181
Clostridium sp. Culture-27	0	0.2	0.373900966
Streptococcus parasanguinis	0	0.2	0.373900966
Gemella haemolysans	0	0.2	0.373900966
Campylobacter sp. 10_1_50	0	0.2	0.373900966
Prevotella aurantiaca	0	0.2	0.373900966
Streptococcus sp. oral strain T1-E5	0	0.2	0.373900966
Dialister sp. E2_20	0	0.2	0.373900966
Cardiobacterium sp. A	0	0.2	0.373900966
Lactuca sativa	0	0.2	0.373900966
Staphylococcus hominis	0	0.2	0.373900966
Staphylococcus succinus	0	0.2	0.373900966
Akkermansia muciniphila	0.25	0	0.391002219
Bifidobacterium pseudolongum	1.75	0	0.391002219
Veillonella tobetsuensis	0.25	0	0.391002219
Staphylococcus aureus	1.25	0	0.391002219
Weissella cibaria	0.25	0	0.391002219
Prevotella pallens	0.25	0	0.391002219
Prevotella salivae	0.25	0	0.391002219
Alloprevotella rava	0.25	0	0.391002219
Eubacterium saphenum	0.25	0	0.391002219
Megamonas funiformis	0.25	0	0.391002219
Megasphaera micronuciformis	0.25	0	0.391002219
Paraprevotella clara	0.25	0	0.391002219
Prevotella IK062	0.25	0	0.391002219
Streptococcus australis	0.25	0	0.391002219
Streptococcus sp. F0442	0.25	0	0.391002219
Clostridium sp. ID4	0.25	6.2	0.391747666
Lactobacillus reuteri	176.25	104.6	0.407845772
Lactobacillus johnsonii	558	376	0.425085487

Genus and species levels of 16S rRNA gene sequencing analysis.

(**a** and **b**) Results of 16S rRNA gene sequencing analysis of the feces of WT (n = 5) and *Gp2*$^{-/-}$ (n = 4) mice. Bacterial species with significant differences are shown in red (unpaired two-tailed Student's *t*-test).

Supplementary Figure 11

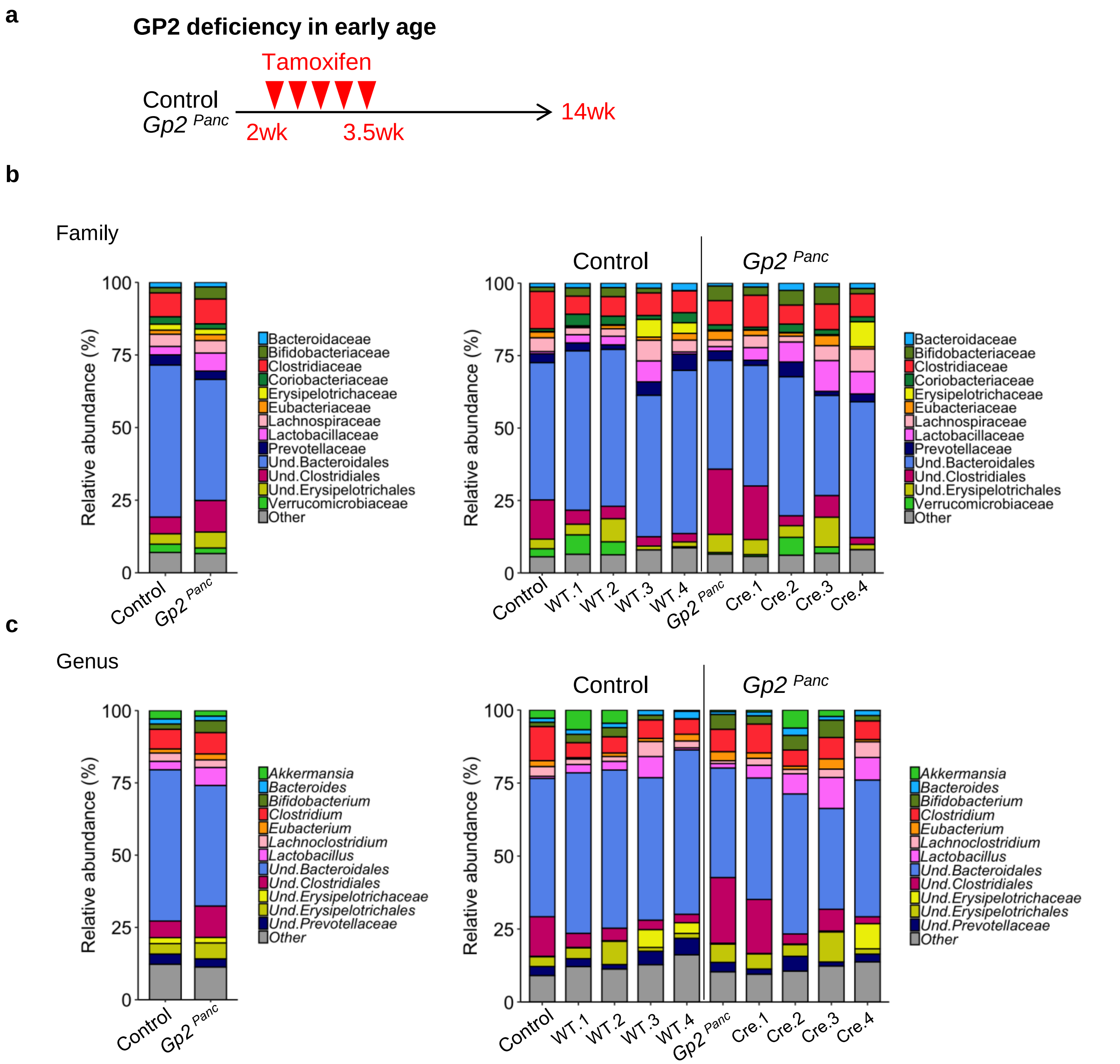

No alteration of commensal microbiota by GP2-deficiency in the early age.

a, Experimental protocol for inducing GP2 deficiency in young mice (2–3.5 weeks old). Feces was collected at week 14. Family- (**b**) and genus-level (**c**) 16S rRNA gene sequencing data are shown as averages for the whole group (left) and for individual mice (right).

Supplementary Figure 12

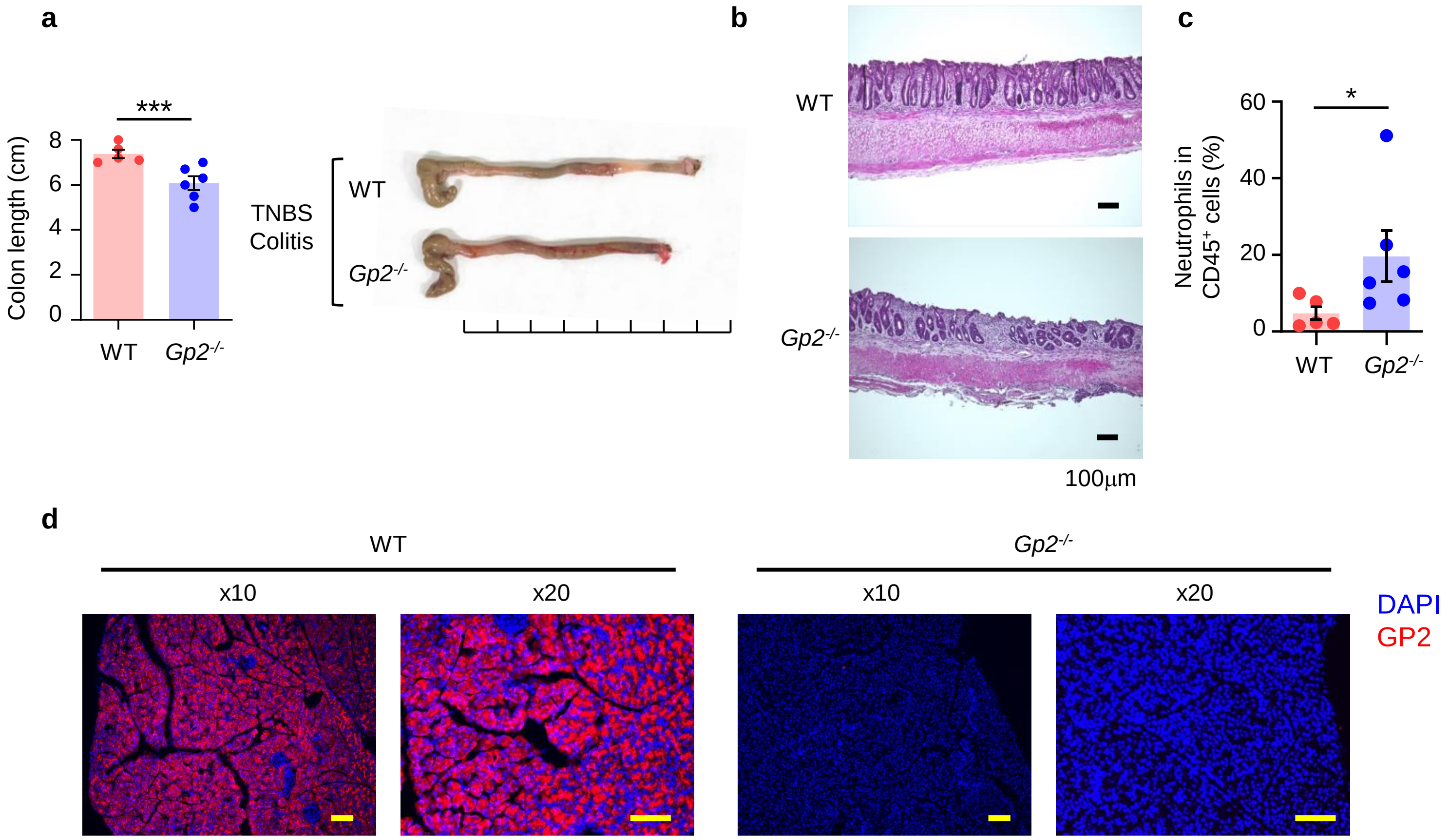

Deletion of systemic GP2 results in severe TNBS-induced colitis.

a, Changes in colon length and photographs of representative colons of WT (n=5) and *Gp2*$^{–/–}$ (n=6) mice with TNBS-induced colitis. ***: p = 0.0065 (two-tailed Mann–Whitney U test). Data are presented as mean values +/- SEM. **b**, Hematoxylin and eosin staining of colon at day 5 of TNBS treatment in wild-type (WT) and *Gp2*$^{–/–}$ mice. Data are representative of two independent experiments. **c**, Percentage of neutrophils in the CD45^{+} cell population in WT (n=5) and *Gp2*$^{–/–}$ (n=6) mice with TNBS-induced colitis was determined by flow cytometry. *: p = 0.0303 (two-tailed Mann–Whitney U test). Data are presented as mean values +/- SEM. **d**, GP2 staining in TNBS-treated WT and *Gp2*$^{–/–}$ mice. Scale bars, 100 µm. Data are representative of two independent experiments. Source data are provided as a Source Data file.

Supplementary Figure 13

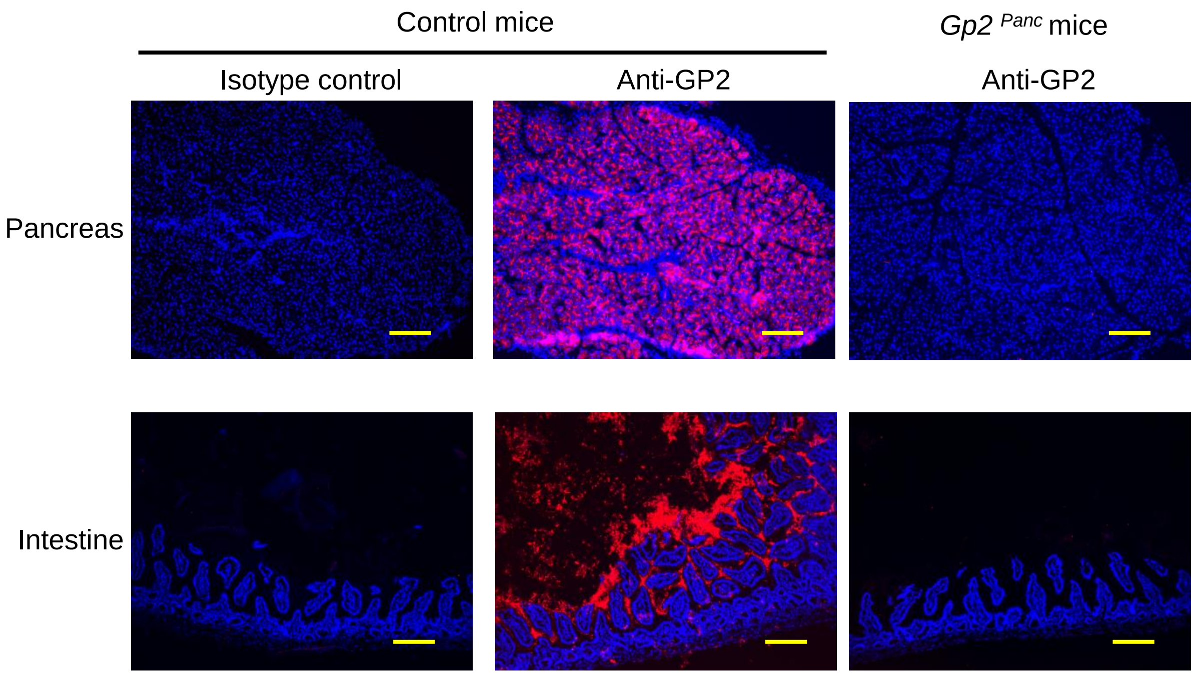

The lack of pancreatic GP2 in tamoxifen treated *Ptfla-cre*^{ETRM}*-Gp2*$^{flox/flox}$ (*Gp2* ^{Panc}) mice. Isotype control or GP2 stained in tamoxifen administered control or *Ptfla-cre*^{ETRM}*-Gp2*$^{flox/flox}$ (*Gp2* ^{Panc}) mice are shown. Represent pictures of pancreas and intestine are shown. Scale bars, 100 μm. Data are representative of three independent experiments.

Supplementary Figure 14

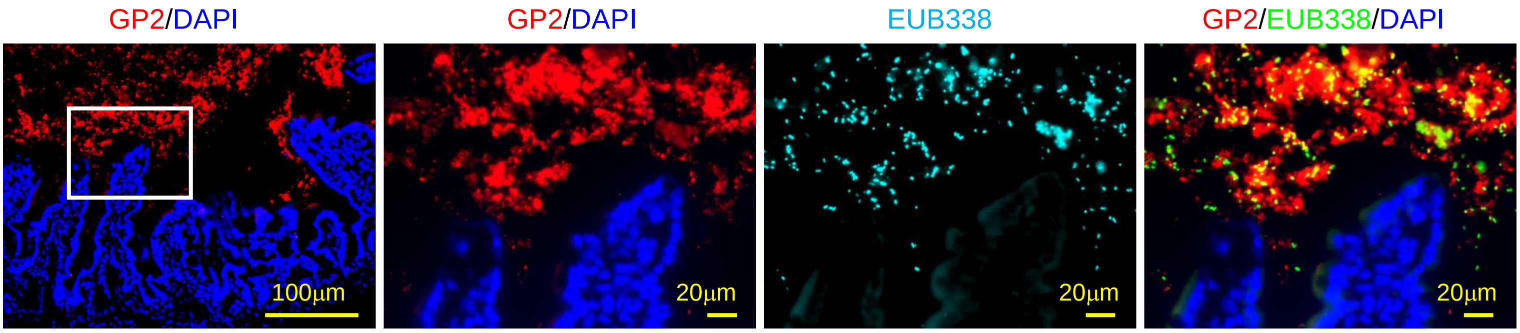

Detection of luminal GP2 and bacteria in small intestine.

Luminal contents of small intestine were stained with GP2 (red) and EUB338 (blue or green). Representative pictures are shown. Data are representative of three independent experiments.

Supplementary Figure 15

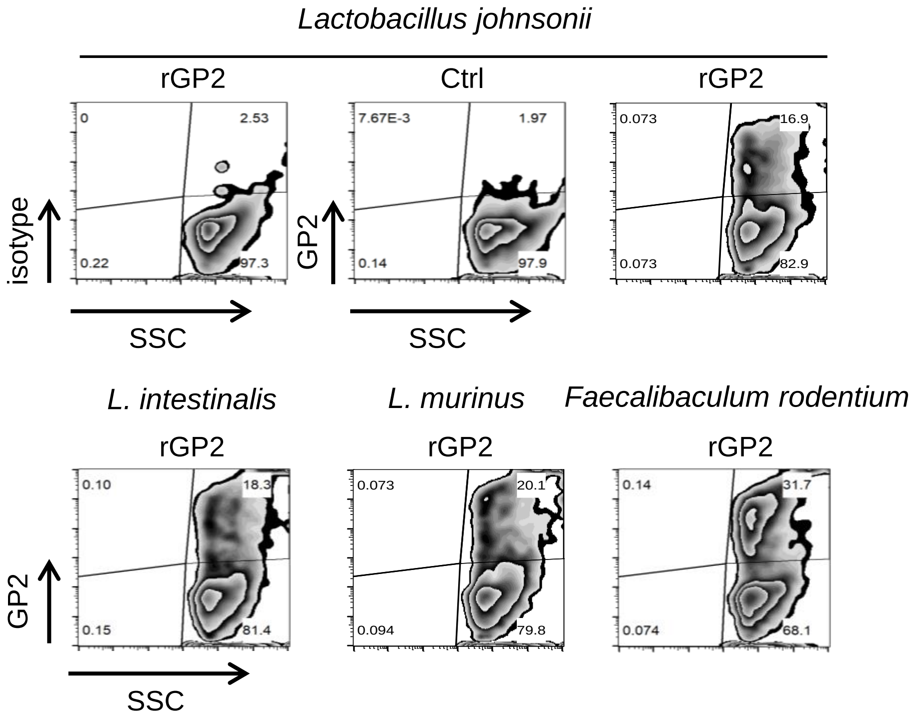

Characterization of GP2-bound commensal bacteria.

a, GP2-bound commensal bacteria were isolated by MACS. Isolated GP2-bound bacteria were cultured (1 x 10^6 CFU) with recombinant mouse GP2 (20 μg) and examined by FACS. Data are representative of three independent experiments.

Supplementary Figure 16

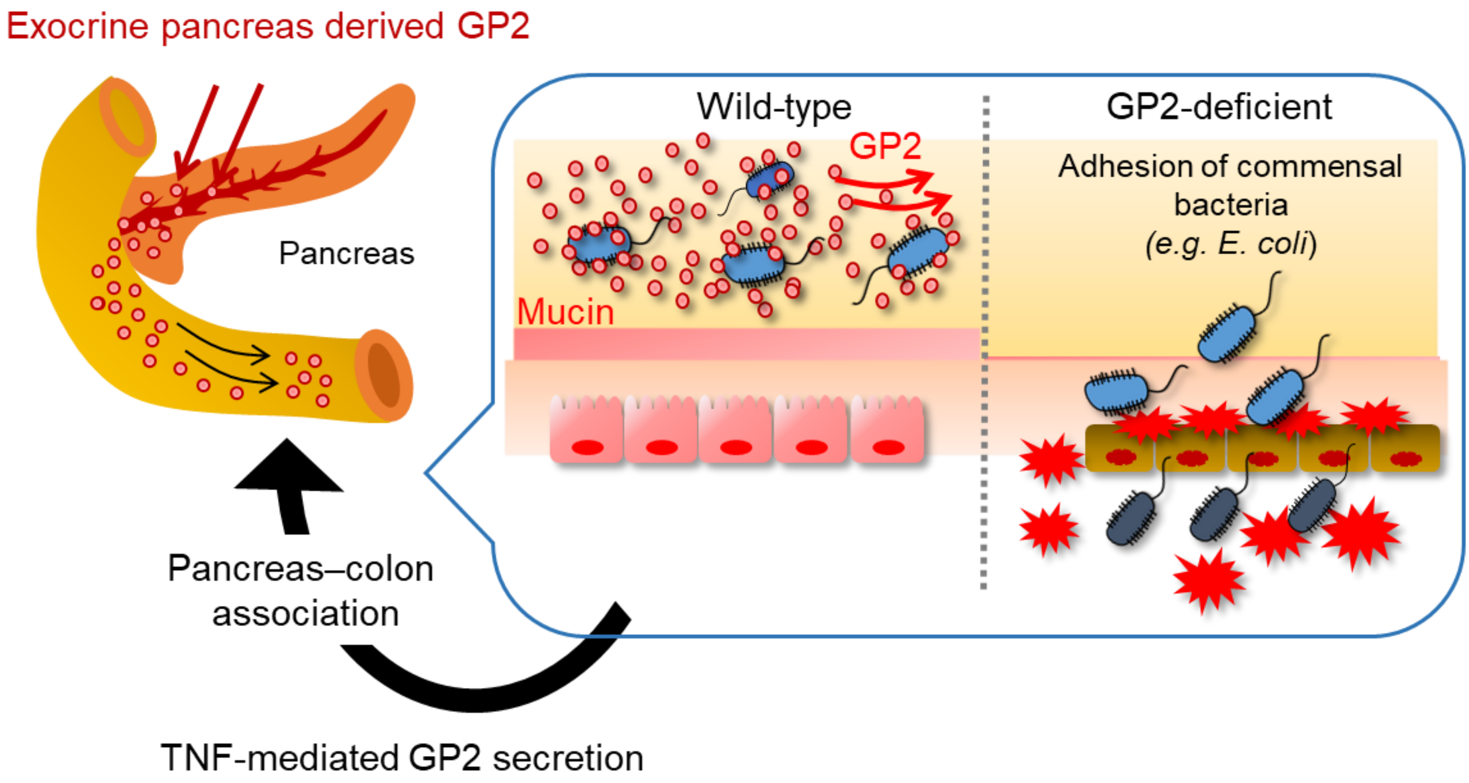

Pancreatic Glycoprotein 2 as a First Line of Defense for Mucosal Protection in Intestinal Inflammation.

Exocrine pancreas derived GP2 bounds to luminal bacteria and regulates bacterial translocation. The scheme of current study has been shown.

Supplementary Figure 17

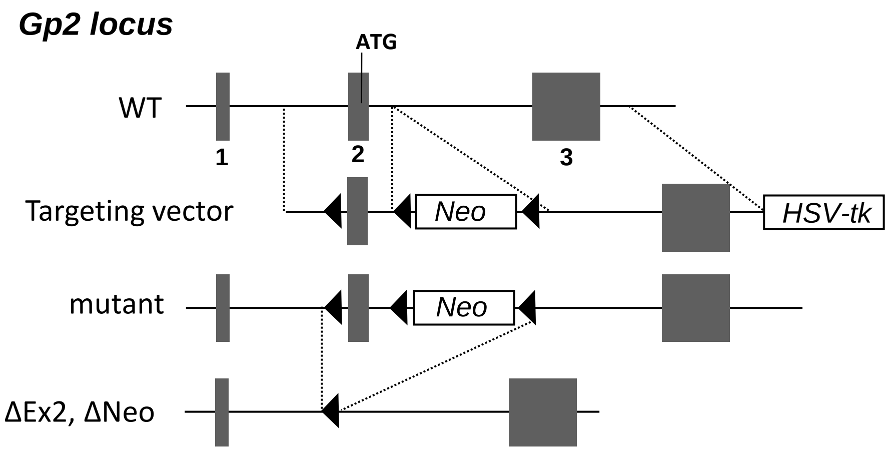

Targeted disruption of the mouse *Gp2* gene by homologous recombination.

Schematic representation of the genomic structure of wild-type mouse *Gp2* gene exons 1-3, the targeting vector, the resultant mutant allele generated by homologous recombination (mutant), and the locus after removal of the region of the genome containing exons 2 and the neomycin resistant gene (ΔEx2, ΔNeo). Exons are indicated by filled boxes.

Supplementary Figure 18

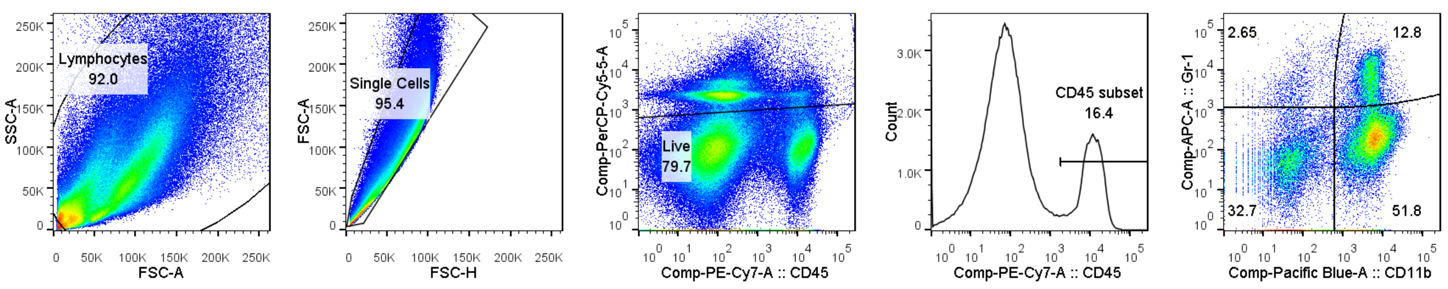

Gating strategy of lamina proprial cell flow cytometry.

The gating strategies for FACS analysis of mouse colon cells are shown.

Supplementary Figure 19

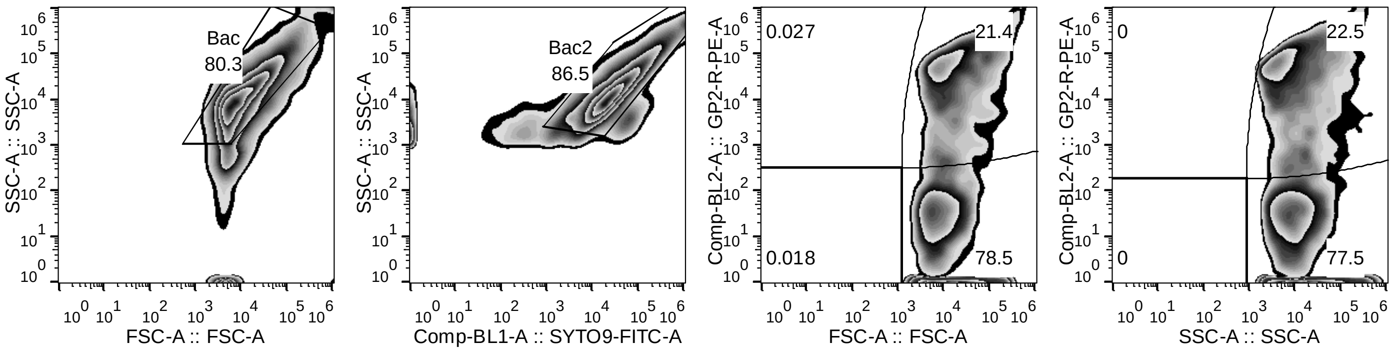

Gating strategy of bacterial flow cytometry.

The gating strategies for FACS analysis of isolated and luminal bacteria are shown.

Supplementary Table

List of primer sequences used for RT-PCR analysis in this study.

Primer Sequences

	**Forward**	**Reverse**
Gapdh	TGTCCGTCGTGGATCTGAC	CCTGCTTCACCACCTTCTTG
Gp2	GATACTTTGGGTTCTTCTCTTATGGT	GCAGCCTGCAGATTCTTCATA
Pnlip	CTGTGGACATTTGCAGTGCT	TGAAGCAGCCGAGTTTGTC
Rab3d	AAGTGTGACCTGGAAGACGAA	GCTGGCCTCAAAGAACTCAA
Rab6a	AATCCGCTGAGGAAATTCAA	TGTCATACATGAATCGGGTGA
Rab8a	TCAAAGCAAAAATGGACAAAAA	TCCACTGTGATCTTGACTCCA
Rab27b	CGGGAAAAACGTGTGGTT	AAGCTGCAGATGTACCTTAAACG
Rap1a	CCCAACGATAGAAGATTCCTACA	GCTCGGTTCCTGCAGTGT
Vamp2	CCAAGCTCAAGCGCAAAT	GGGATTTAAGTGCTGAAGTAAACG
Vamp3	TCGCAGTTTGAAACAAGTGC	ATCCCTATCGCCCACATCTT
Vamp8	CAGAATGTGGAGCGGATCTT	TGGGACGTTGTCTTGAAGTG
Stx3	CCGTTGGGCTGAAATAAGAG	AAGCTGGAGTGAAAGCTGGT
Stx7	GAGTTCGTTGCTCGAGTGC	TTCTTTTGAGCTGTCTTCAGGA
Try5	TCCCCTACCAGGTGTCTCTG	TCACTTGGATGCGGGTTT
Plcxd2	AGGAGGCCTTTGGGAACA	GAAGGGACAGTGGTAGAAAATGA
Plch1	CTGAAGCAGCAAAAGACACG	GCTCCCTTTCTTCTGTCATCC
Cpa1	CGCAAAACTCGATCACACAC	CTACTAGCTCCGGGCATCC
Cpa2	CGAGAGTGGGTTACGCAAG	TCCCATAATCAGACGCAATCT
Reg2	CTGCCAACCGTGGTTATTG	GGCTCTGAACTTGCAGACAA
Reg3a	ATTGGGCTCCATGATCCA	AGATAATTCAGCACATCGGAGTT
Reg3b	TGGATTGGGCTCCATGAC	TCATCACGTCATTGTTACTCCA
Reg3g	ACCATCACCATCATGTCCTG	GGCATCTTTCTTGGCAACTT
