# Supplementary material for: Pancreatic glycoprotein 2 is a first line of defense for mucosal protection in intestinal inflammation
Source: Nat Commun. 2021 Feb 16;12:1067. doi: 10.1038/s41467-021-21277-2 (PMC7887276; doi:10.1038/s41467-021-21277-2)
Supplement: Supplementary file 3 — Reporting Summary [file 41467_2021_21277_MOESM3_ESM.pdf]

nature research

Corresponding author(s): Hiroshi Kiyono

Last updated by author(s): Dec 14, 2020

Reporting Summary

Nature Research wishes to improve the reproducibility of the work that we publish. This form provides structure for consistency and transparency in reporting. For further information on Nature Research policies, see our Editorial Policies and the Editorial Policy Checklist.

Statistics

For all statistical analyses, confirm that the following items are present in the figure legend, table legend, main text, or Methods section.

n/a	Confirmed	
☐	☒	The exact sample size (n) for each experimental group/condition, given as a discrete number and unit of measurement
☐	☒	A statement on whether measurements were taken from distinct samples or whether the same sample was measured repeatedly
☐	☒	The statistical test(s) used AND whether they are one- or two-sided<br>*Only common tests should be described solely by name; describe more complex techniques in the Methods section.*
☐	☒	A description of all covariates tested
☐	☒	A description of any assumptions or corrections, such as tests of normality and adjustment for multiple comparisons
☐	☒	A full description of the statistical parameters including central tendency (e.g. means) or other basic estimates (e.g. regression coefficient) AND variation (e.g. standard deviation) or associated estimates of uncertainty (e.g. confidence intervals)
☐	☒	For null hypothesis testing, the test statistic (e.g. F, t, r) with confidence intervals, effect sizes, degrees of freedom and P value noted<br>*Give P values as exact values whenever suitable.*
☒	☐	For Bayesian analysis, information on the choice of priors and Markov chain Monte Carlo settings
☒	☐	For hierarchical and complex designs, identification of the appropriate level for tests and full reporting of outcomes
☒	☐	Estimates of effect sizes (e.g. Cohen's d, Pearson's r), indicating how they were calculated

Our web collection on statistics for biologists contains articles on many of the points above.

Software and code

Policy information about availability of computer code

Data collection	FACS CANTO (BD Bioscience), FACS ATTUE (Thermo Fisher Scientific), BZ-9000 (Keyence)
Data analysis	Flow Jo (Treestar Inc., V10), Graphpad (Prism v6 and v8)

For manuscripts utilizing custom algorithms or software that are central to the research but not yet described in published literature, software must be made available to editors and reviewers. We strongly encourage code deposition in a community repository (e.g. GitHub). See the Nature Research guidelines for submitting code & software for further information.

Data

Policy information about availability of data

All manuscripts must include a data availability statement. This statement should provide the following information, where applicable:

- Accession codes, unique identifiers, or web links for publicly available datasets
- A list of figures that have associated raw data
- A description of any restrictions on data availability

The dataset generated and analyzed during the current study are available from the corresponding authors upon reasonable request. Metagenomic 16S rRNA sequencing data have been deposited in the DNA Data Bank Japan (DDBJ) under the accession number “PRJDB10865” http://trace.ddbj.nig.ac.jp/BPSearch/bioproject?acc=PRJDB10865 and “PRJDB10886” http://trace.ddbj.nig.ac.jp/BPSearch/bioproject?acc=PRJDB10886. The source data underlying Figs. 2b, c, e, f, 3d, f, g, I, 4a, c-e, I, j, 5c, d, 6b, 6c, 7, b, c, e, 8a, b, f, g, and Supplementary Figs. 3b, 6a, b, 7, 12 a, c, are provided as a Source Data file.

Field-specific reporting

Please select the one below that is the best fit for your research. If you are not sure, read the appropriate sections before making your selection.

- [x] Life sciences
- [ ] Behavioural & social sciences
- [ ] Ecological, evolutionary & environmental sciences

For a reference copy of the document with all sections, see nature.com/documents/nr-reporting-summary-flat.pdf

Life sciences study design

All studies must disclose on these points even when the disclosure is negative.

Sample size	We did not performed sample-size calculation. The sample size was empirically started at 3 or 5, and was increased at the next experiment when there was no significant difference. Ultimately at least two independent experiments were conducted to draw conclusions.
Data exclusions	No data was excluded in all our experiments.
Replication	Experiments were repeated and with sufficient animals per group to demonstrate statistical significance. All experiments were reliable reproduced.
Randomization	Animals were randomly allocated for examination.
Blinding	Blinding was not performed since the data analysis was strictly quantitative and not subjective and the data collection and analysis were performed with quantitative instruments to maintaining objectivity.

Reporting for specific materials, systems and methods

We require information from authors about some types of materials, experimental systems and methods used in many studies. Here, indicate whether each material, system or method listed is relevant to your study. If you are not sure if a list item applies to your research, read the appropriate section before selecting a response.

Materials & experimental systems

n/a	Involved in the study	
☐	☒	Antibodies
☐	☒	Eukaryotic cell lines
☒	☐	Palaeontology and archaeology
☐	☒	Animals and other organisms
☐	☒	Human research participants
☒	☐	Clinical data
☒	☐	Dual use research of concern

Methods

n/a	Involved in the study	
☒	☐	ChIP-seq
☐	☒	Flow cytometry
☒	☐	MRI-based neuroimaging

Antibodies

Antibodies used	anti-mGP2 (MBL, 2F11-C3, #D278-3, 1:200) , Alexa 555-conjugated anti-rat IgG (BioLegend, Poly4054, #405420, 1:200), isotype control (rat IgG2a, BioLegend, RTK2758, #400501, 1:100), isothiocyanate (FITC)-conjugated UEA-1 (Vector Laboratories, # FL-1061, 1:100), DAPI (4',6-diamidino-2-phenylindole; Dojindo, #D523, 1:1000), anti-CD16/32 antibody (Fc block; BD Biosciences, 2.4G2, #553141, 1:500), (PE)-conjugated anti-podoplanin (Biolegend, 8.1.1, #127410, 1:1000), Alexa 647-conjugated anti-EpCAM (Biolegend, G8.8, #118211, 1:500), PE-Cy7-conjugated anti-CD90.2 (Biolegend, 5302.1, #140410, 1:500), Pacific Blue-conjugated anti-CD45 (BioLegend, R30-F11, #103126, 1:100), Gr-1 (Biolegend, RB6-8C5, #108412, 1:500), CD11b (Biolegend, M1/70, #101262 and # 101224, 1:500), anti-MUC2 antibody (Santa Cruz Biotechnology, sc15334, Lot#F1915, 1:50), DyLight 488-conjugated anti-rabbit IgG (BioLegend, Poly4046, #406404, 1:200), anti-mouse Ig(H+L) (1 ug/mL; Southern Biotech #1010-01), anti-mouse IgA (Southern Biotech #1040-05, 1:4000), anti-mouse IgG (Southern Biotech #1040-05, 1:4000), anti-mouse IgM (Southern Biotech #1020-05, 1:4000).
Validation	All antibodies and kits came from commercial vendors, and were validated by the manufacturers on their websites.<br>The specificity of anti-GP2 antibodies was confirmed by Western blotting and immunohistochemistry of tissues from GP2 knockout mice.

Eukaryotic cell lines

Policy information about cell lines

Cell line source(s)	HEK293T cells were purchased from ATCC.
Authentication	human embryonic kidney (HEK293T) cells were purchased from ATCC and cultured according to recommendations. Cells used

	for transiently transfected with the rGP2 expression vectors.
Mycoplasma contamination	Cells were tested negative for mycoplasma.
Commonly misidentified lines (See ICLAC register)	No cell lines used are listed in the ICLAC database.

Animals and other organisms

Policy information about studies involving animals; ARRIVE guidelines recommended for reporting animal research

Laboratory animals	C57BL/6J mice were purchased from CLEA Japan, Inc. (Tokyo, Japan) or Japan SLC, Inc. (Shizuoka, Japan). C57BL/6J mice 8- to 10-week-old male C57BL/6 mice were used in this study. Vil1-cre (#004586), Ptfla-creERTM (#019378), Rosa26-tdTomato (#007914) mice were purchased from Jackson Laboratory. Col1a2-GFP mice were provided by Prof. Yutaka Inagaki (Higashiyama, R. et al. Negligible contribution of bone marrow-derived cells to collagen production during hepatic fibrogenesis in mice. Gastroenterology 137, 1459-1466, 2009). Gp2flox/flox mice were constructed by Prof. Koji Hase and Dr. Hiroshi Ohno and construction is mentioned in the Supplementary Figure 17 and Method section . Gp2–/– mice have been generated (Michaels, M. A. et al. Pancreatic Autoantibodies Against CUZD1 and GP2 Are Associated with Distinct Clinical Phenotypes of Crohn's Disease. Inflammatory Bowel Diseases 21, 2864-2872, 2015). 8 weeks- old male mice were used to this study, except the tamoxifen treatment analysis, which 2 weeks old mice were used in data of Supplmentary Figure 11.
Wild animals	This study did not involve wild animals.
Field-collected samples	This study did not involve samples collected from the field.
Ethics oversight	All mice except germ-free mice were maintained under specific-pathogen-free conditions at the experimental animal facility of the Institute of Medical Science, The University of Tokyo and Chiba University, Japan. All experiments were approved by the Animal Care and Use Committee of the University of Tokyo and Chiba University.

Note that full information on the approval of the study protocol must also be provided in the manuscript.

Human research participants

Policy information about studies involving human research participants

Population characteristics	An IBD-specializing gastroenterologist-confirmed diagnosis of inflammatory bowel disease (except healthy controls) were recruited, excluding those who were pregnant, had other autoimmune or inflammatory diseases (except for extra-intestinal manifestations of IBD), had a malignancy. All clinical data for subjects was current at the time of sample collection.An IBD-specializing gastroenterologist-confirmed diagnosis of inflammatory bowel disease (except healthy controls) were recruited, excluding those who were pregnant, had other autoimmune or inflammatory diseases (except for extra-intestinal manifestations of IBD), and had a malignancy. IBD patients were diagnosed according to the endoscopic, radiologic, histologic, and clinical criteria provided by the International Organization for the Study of Inflammatory Bowel Disease (#1,#2). All clinical data for subjects was current at the time of sample collection. #1. Podolsky DK. Inflammatory bowel disease (1). N Engl J Med. 1991;325:928-937, #2. Podolsky DK. Inflammatory bowel disease (2). N Engl J Med. 1991;325:1008-1016
Recruitment	Patients in this study were recruited from Osaka University Hospital (Osaka, Japan). There were no self-selection and consented patients were recruited if they presented with inflamed mucosa.
Ethics oversight	The experiments were approved by the human ethical committee of Osaka University Hospital and The University of Tokyo, and all tissues were sampled with written informed consent.

Note that full information on the approval of the study protocol must also be provided in the manuscript.

Flow Cytometry

Plots

Confirm that:

- [x] The axis labels state the marker and fluorochrome used (e.g. CD4-FITC).
- [x] The axis scales are clearly visible. Include numbers along axes only for bottom left plot of group (a 'group' is an analysis of identical markers).
- [x] All plots are contour plots with outliers or pseudocolor plots.
- [x] A numerical value for number of cells or percentage (with statistics) is provided.

Methodology

Sample preparation	Large intestines were dissected into short segments and stirred at 37 degree in prewarmed RPMI 1640 containing 2% FCS and 0.5 mM EDTA for 15 min, followed by vigorous shaking for 15 s. This process was repeated twice. The isolated de-epithelialized parts of whole colon were digested in 1.25 mg/ml collagenase at 37degree (WAKO) for 20-30 min for 3 times. The collected mononuclear cells were subsequently filtered through a 40 or 70µm cell strainer (BD Biosciences) and examined further.

	Fecal bacterial samples were analyzed by spectrophotometer, and 1x 10^7 cells were fixed by 4% paraformaldehyde at 4 degree for 3 h. After centrifuge and discard supernatants, bacteria were resuspended and examined further.
Instrument	FACS Canto (BD), FACS Attune (Thermo fisher Scientific), FACS Aria II (BD)
Software	FACS Diva was used to collect data and FlowJo to analyze data. For cell sorting for qPCR.
Cell population abundance	For acquiring data for cellular and bacterial phenotyping, at least 5000-10000 cells were included in the stopping gate.
Gating strategy	Flow cytometry: First, lymphocytes were gating using the SSC-A vs. FSC-A plot. Then, single cells were gated using the FSC-H vs. FSC-A plot, and live cells were gated as 7-AAD negative. Additional information of gating strategies for the experiments were shown in the Supplementary Figures 18 and 19.

[x] Tick this box to confirm that a figure exemplifying the gating strategy is provided in the Supplementary Information.
